# Correction: Decoding the language of microbiomes using word-embedding techniques, and applications in inflammatory bowel disease

**DOI:** 10.1371/journal.pcbi.1008423

**Published:** 2020-11-02

**Authors:** Christine A. Tataru, Maude M. David

In Figs 4 and 5, there are infographics included to clarify the workflow, however, the labels on these graphics are incorrect. The authors have provided a corrected version of Figs 4 and 5 here.

**Fig 4:**
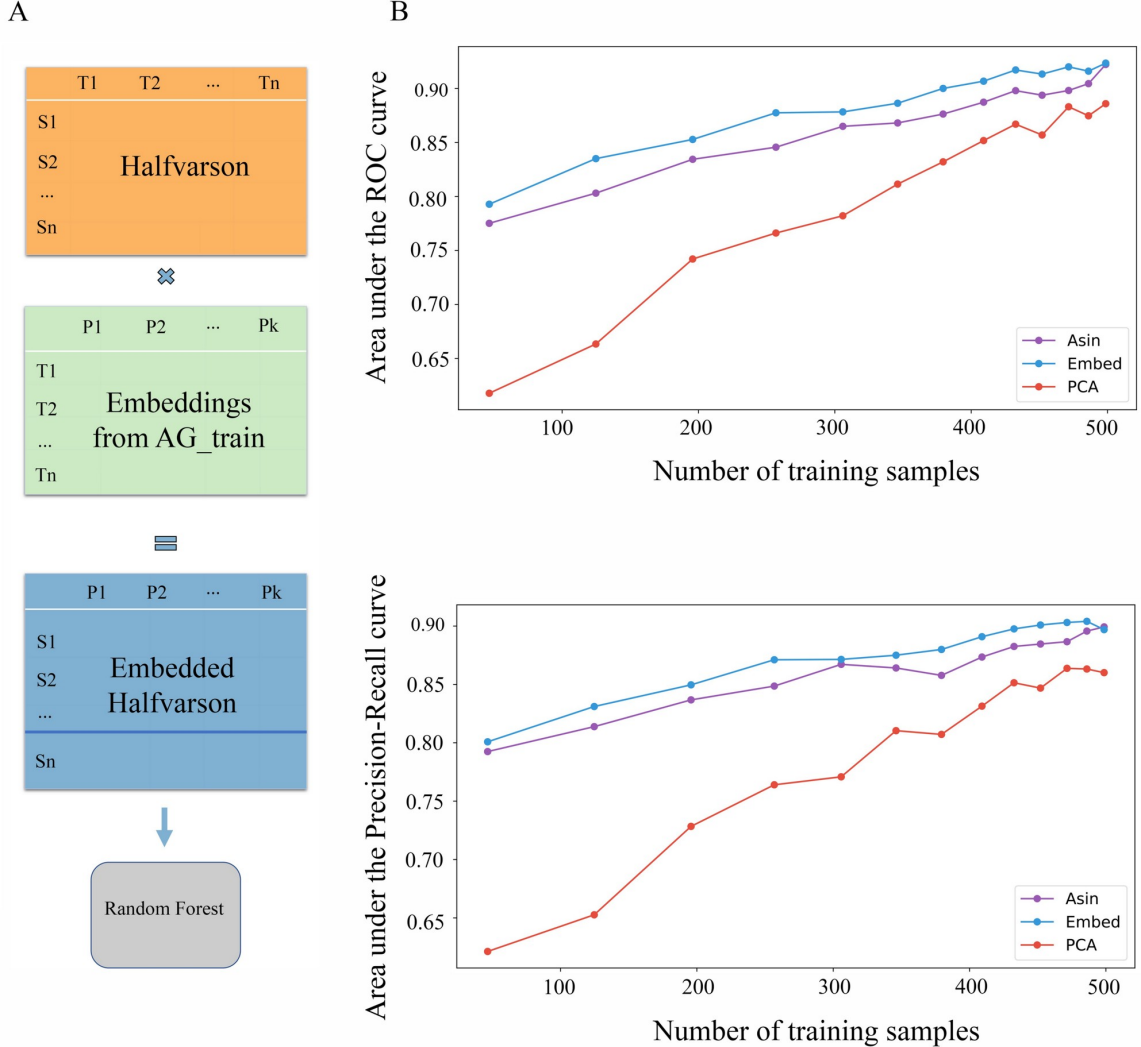
Embeddings were trained on American Gut data, and the predictive models were trained and tested on Halfvarson dataset (A). Transforming microbiome data into GloVe embedding space (100 features) prior to model training produces more accurate models than using ASVs (26,251 features) (B).

**Fig 5:**
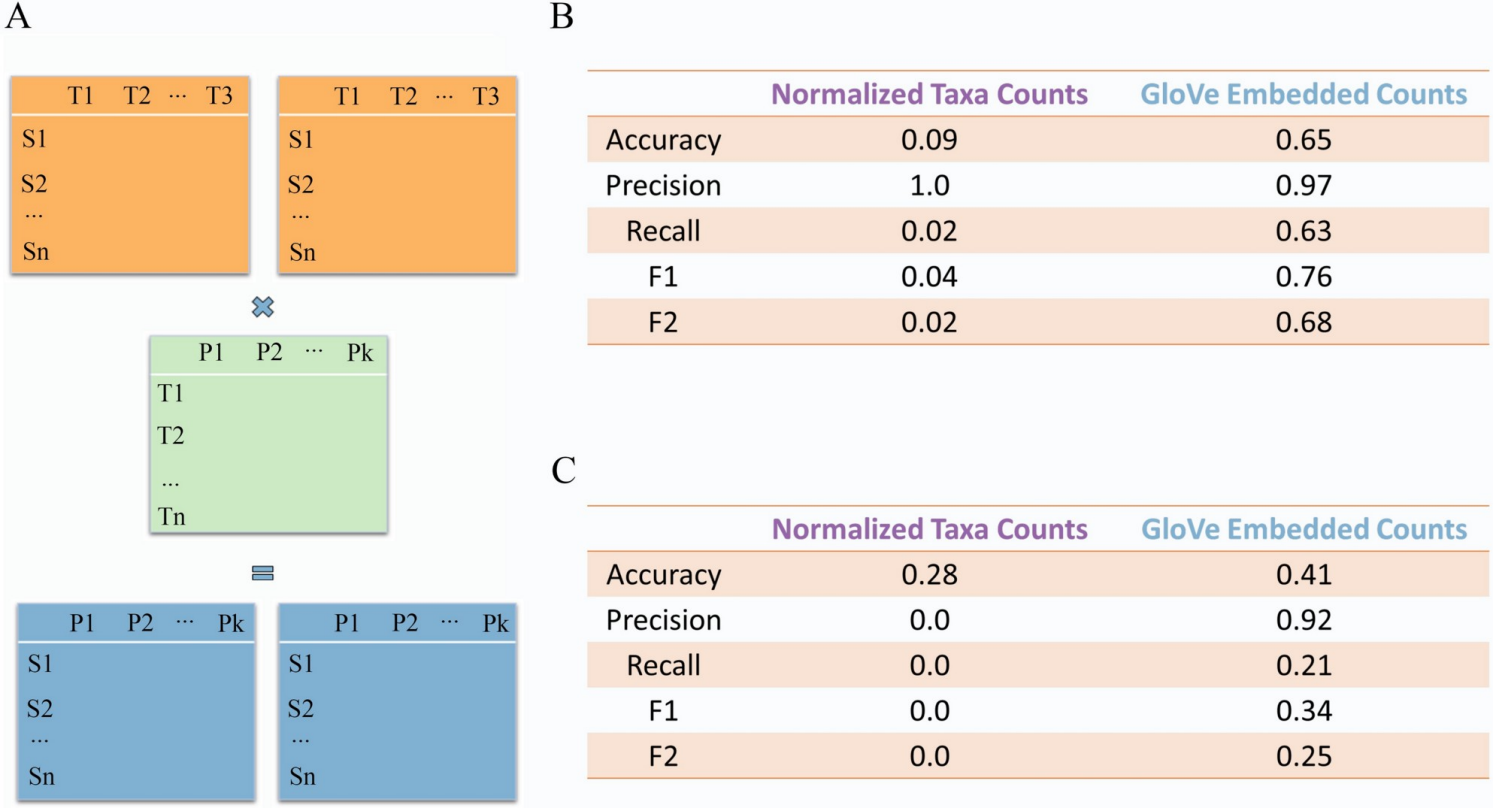
Two models, one embedding-based and one ASV-based, were trained on American Gut data and tested on two independent query datasets (A). Embedding-based models outperform ASV-based models significantly when testing on Halfvarson dataset (B) and Schirmer dataset (C).
