# Supplementary figures and images for: Decoding the language of microbiomes using word-embedding techniques, and applications in inflammatory bowel disease
Source: PLoS Comput Biol. 2020 May 4;16(5):e1007859. doi: 10.1371/journal.pcbi.1007859 (PMC7244183; doi:10.1371/journal.pcbi.1007859)

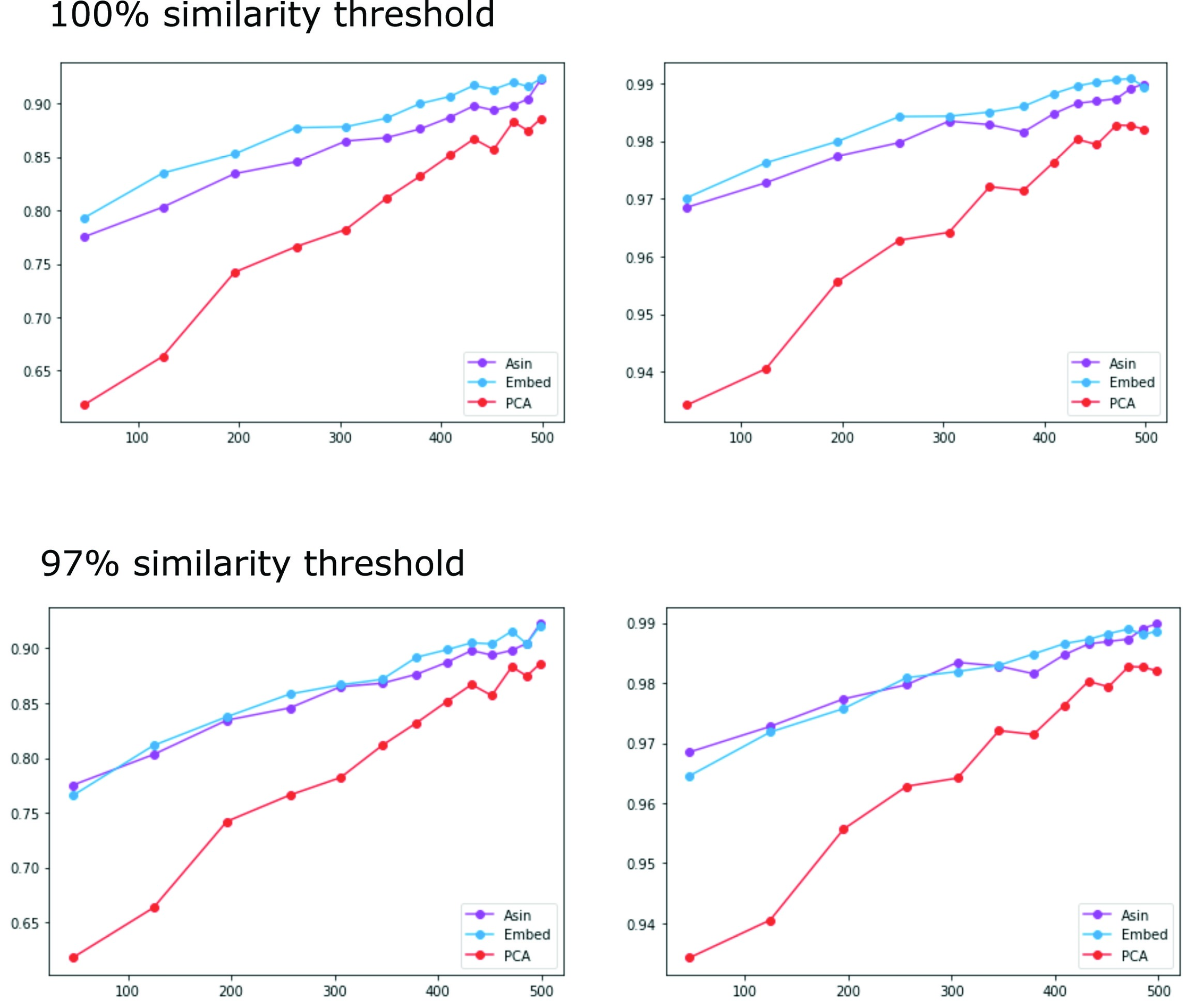

Supplement: S1 Fig — Embeddings were trained on American Gut data, and the predictive models were trained and tested on Halfvarson dataset. Transforming microbiome data into GloVe embedding space (blue, 100 features) prior to model training produces more accurate models than using ASVs (purple, 26,251 features). ASVs from the either dataset were matched to the embedding transformation matrix if sequences were 100% (A) or 97% (B) similar. (TIF) [file pcbi.1007859.s006.tif]

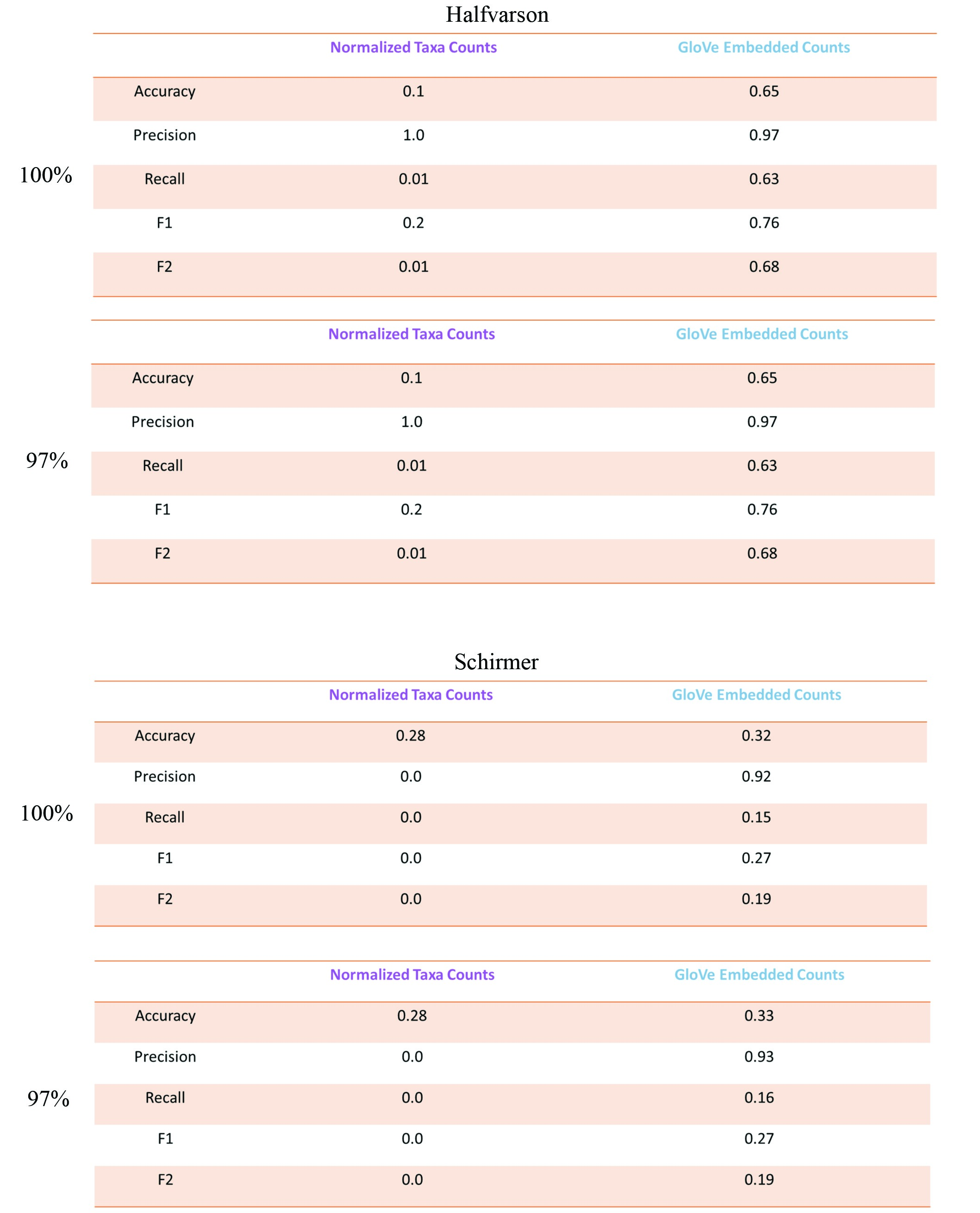

Supplement: S2 Fig — Two models, one embedding-based and one ASV-based, where trained on American Gut data and tested on Halfvarson dataset (A) and Schirmer dataset (B). Embedding-based models outperform ASV-based models significantly. ASVs were matched to the embedding transformation matrix if sequences were 100% or 97% similar, as indicated. (TIF) [file pcbi.1007859.s007.tif]

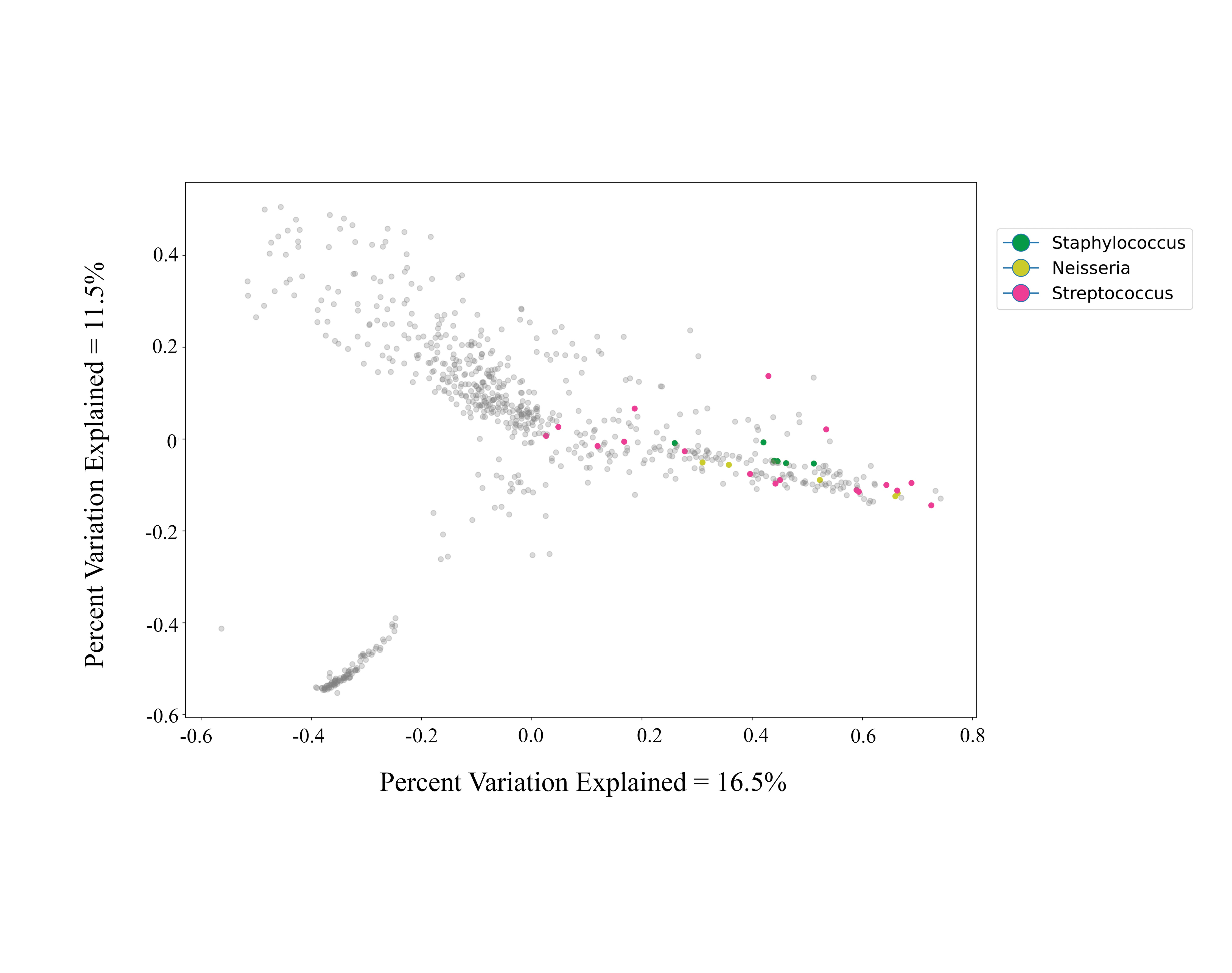

Supplement: S3 Fig — PCoA ordination of randomly selected 5000 ASVs using correlation of normalized counts. 3 genera, Staphylococcus, Neisseria, and Streptococcus that have similar embedding patterns likely co-occur directly. (TIF) [file pcbi.1007859.s008.tif]

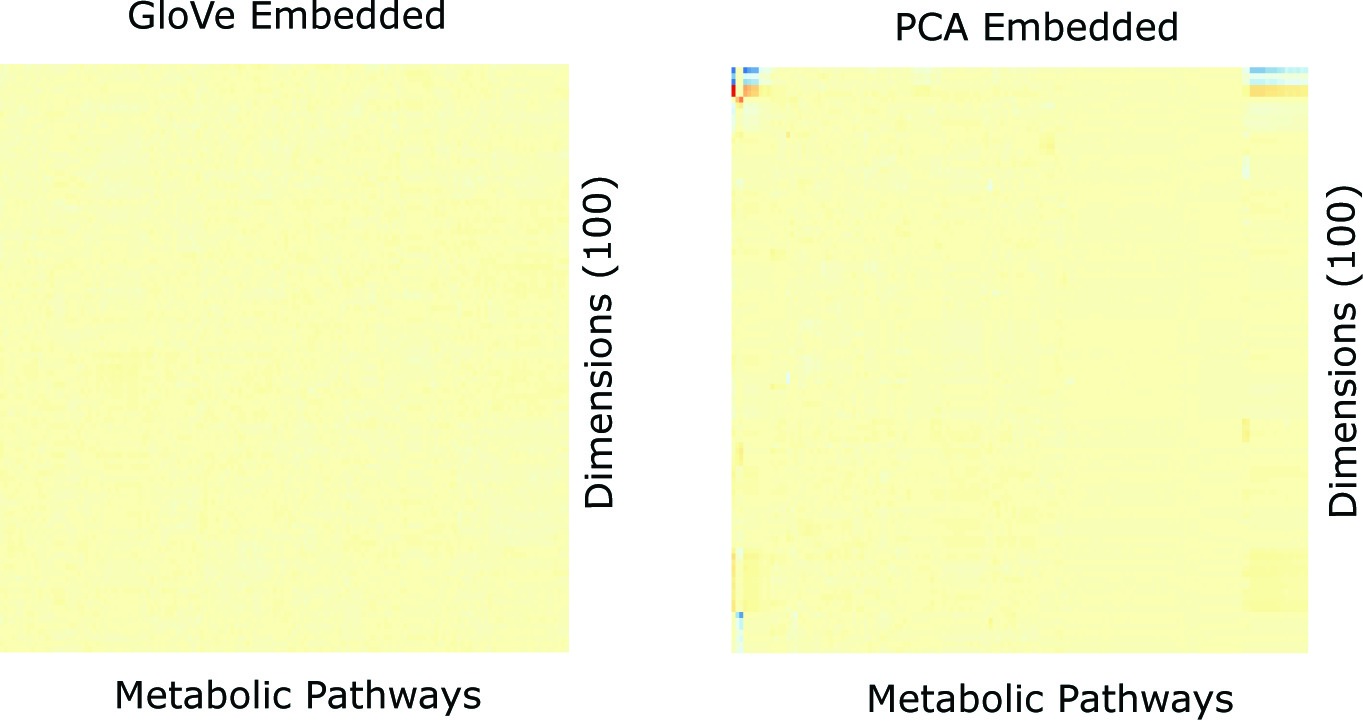

Supplement: S4 Fig — Heatmaps showing correlations between dimensions in transformed space and one null annotated metabolic pathway table. We see far fewer and less dramatic correlations between transformed data and metabolic pathways when the pathway table has been shuffled. (TIF) [file pcbi.1007859.s009.tif]

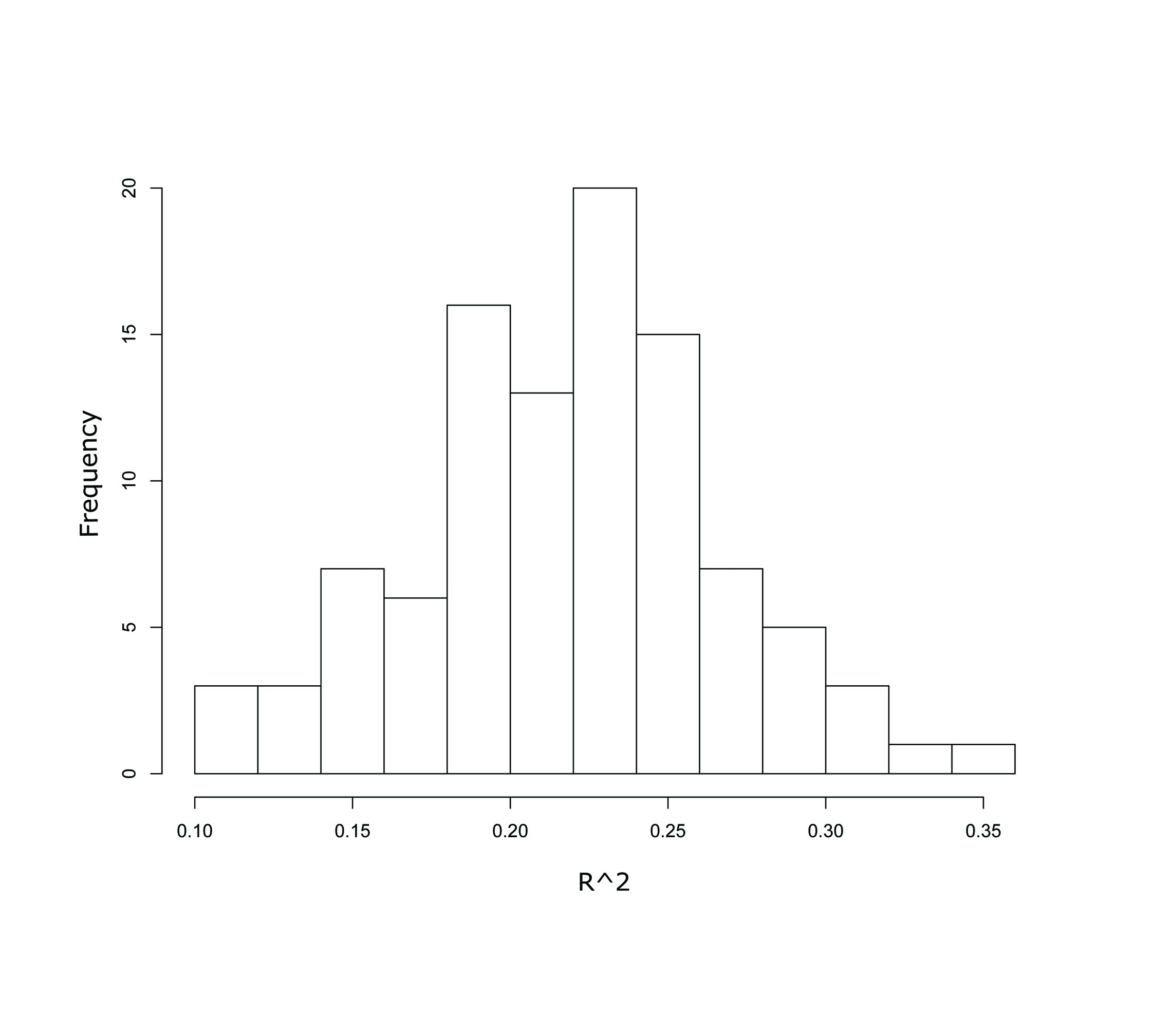

Supplement: S5 Fig — Histogram depicting the percent variance of properties explainable by annotated metabolic pathways. Most properties are less than 25% explained by pathways, and no property is more than 36% explained. (TIF) [file pcbi.1007859.s010.tif]
