# Supplementary material for: Decoding the language of microbiomes using word-embedding techniques, and applications in inflammatory bowel disease
Source: PLoS Comput Biol. 2020 May 4;16(5):e1007859. doi: 10.1371/journal.pcbi.1007859 (PMC7244183; doi:10.1371/journal.pcbi.1007859)
Supplement: Attachment — Submitted filename: answer_reviewers_letter-merged.pdf [file pcbi.1007859.s011.pdf]

Maude M David, PhD
Assistant Professor
Dept. of Microbiology
Oregon State University
226 Nash Hall , Corvallis, Oregon
97331
P: (541)737-0629
E: maude.david@oregonstate.edu

Dr. Grilli, Dr. Allesina,

Thank you very much for your feedback on our paper. We have taken care to address the reviewers' valuable comments and provide a point-by-point response below.

We hope you find our manuscript sufficiently improved and now suitable for publication.

Sincerely,

Christine Tataru   Maude M. David

* Another idea to remedy Fig. 6 is to hierarchically cluster the OTU embeddings and see how well that tree reconciles (similarity metrics between the two trees) with the phylogenetic tree. IE: there are many ways to better test the idea in Fig. 6.

Thank you for this great suggestion, we agree this is a good way to show a phylogenetic relationship. We have replaced figure 6 and associated text to describe a permutation test comparing tree structures between phylogenetic trees and hierarchically clustered ASVs based on their property vectors. We have also emphasized in the text that the intention was to demonstrate that while there is some relationship between phylogenetic and property metrics, it does not seem that phylogenetics is the main driver of the property vectors.

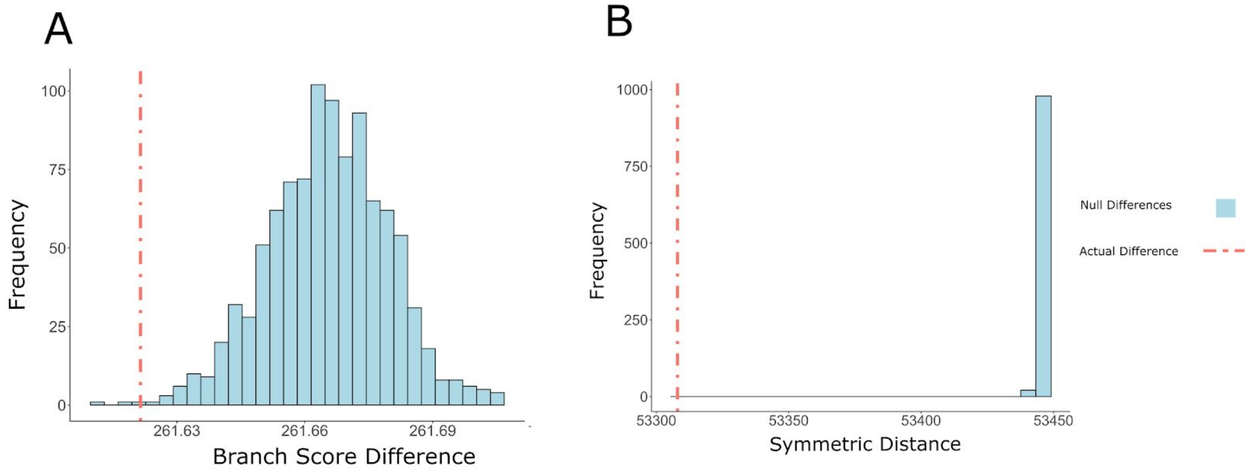

* The biggest aspect from this paper is that the method is learning from all the co-occurrence patterns but never really revealing the learned patterns. There is much interest in knowing which species are actually co-occurring: https://journals.plos.org/ploscompbiol/article?id=10.1371/journal.pcbi.1002606

Using the OTU probabilities and ratios, can the authors shed light on common patterns to certain sample-associated metadata?

- Thank you for your feedback. We also agree, given that in the presented framework, a property is defined as a co-occurrence pattern it would be of interest to shed some light on these associations. To do so, we constructed a taxa-taxa co-occurrence network based on differences in property space. We then calculated the similarity of each ASV to each other ASV in property space, and finally averaged the distances per phylum to dictate edge weight in the network below. We also subsetted to include only the most common phylum. The resulting network did not allow us to extract new information and is difficult to read, even when subsetting to include only the most common phylum (see figure below). Therefore we decided not to include it as a figure in the paper.

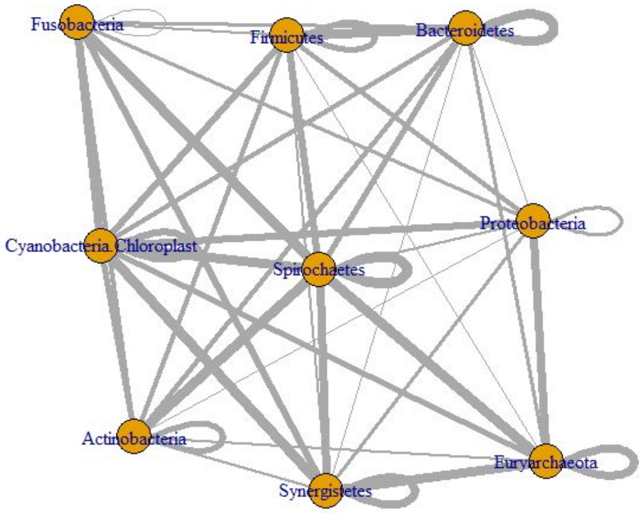

* Fig. 6 is highly confusing, and Section 2.6 can be improved. The authors plot the cosine distances between embedded-vector OTUs vs. their tip-to-tip branch phylogenetic distance. If well correlated, one would see that as the cosine distance increased, the phylogenetic distance would increase. However, this is not the case. We see two islands and not much explanation about why this is in Section 2.6. Why not plot the actual OTU embeddings – could use T-SNE for visualization? Then, it is possible to visualize the distances in the embedding space. The OTUs could then be color-coded via their genus/species/order/phylum level labels to show taxonomic clusterings.

We thank the reviewers for this comment, and agree that this is difficult to read. We've attempted to follow the reviewers' comments and plot the embedding space using both T-SNE (first figure below) and PCA (second figure below), but unfortunately the resulting plot is very difficult to interpret due to the high number of ASVs (26726), which make the plot largely unreadable. We've edited the legend of this figure to explain better that the phylogeny and position in embedding space are only weakly related, and since this figure is not the main focus of our paper and seems to add confusion, we propose to move this figure (contour plot) to be Supplementary Figure 3.

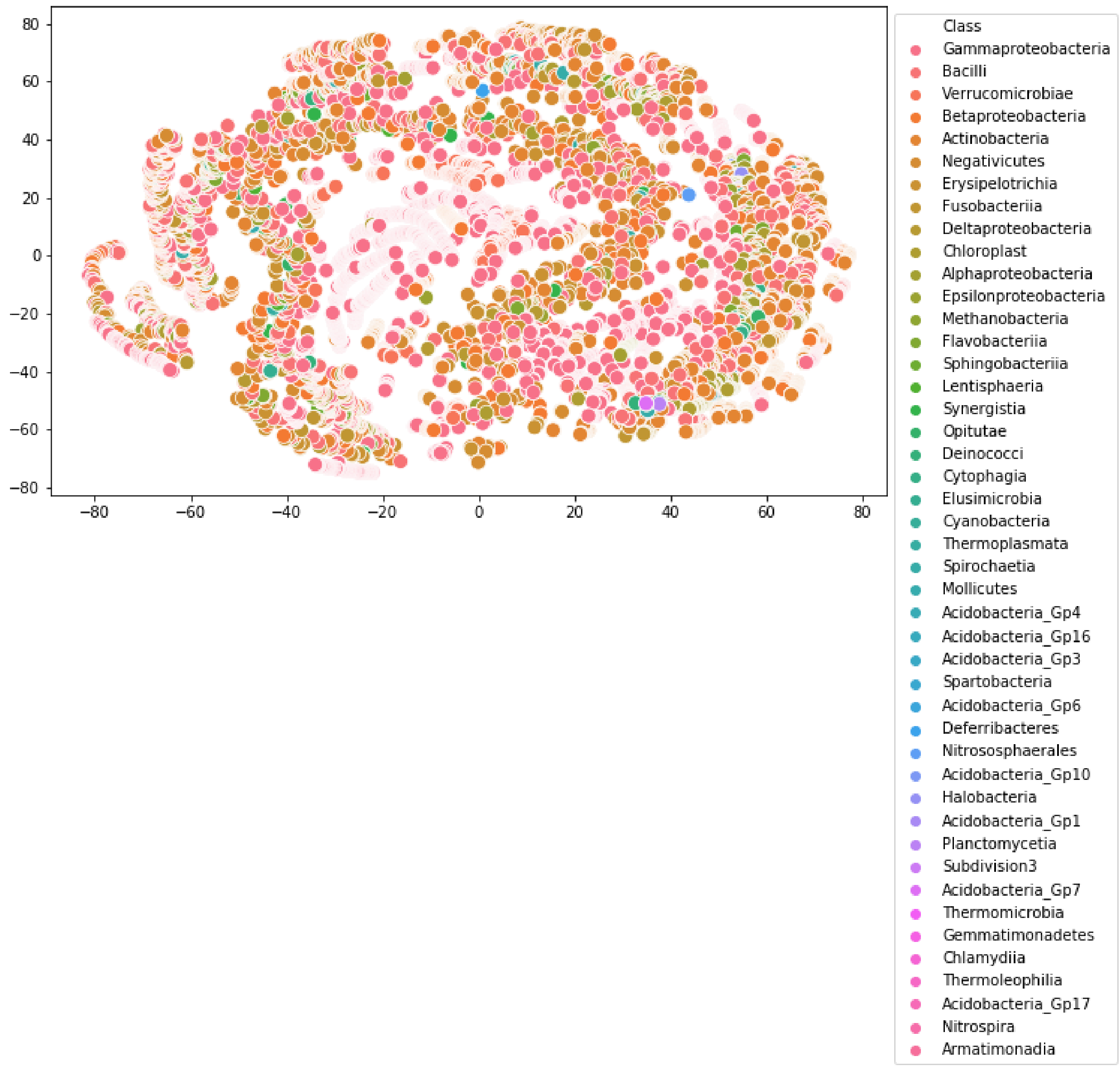
Class
Gammaproteobacteria
Bacilli
Verrucomicrobiae
Betaproteobacteria
Actinobacteria
Negativicutes
Erysipelotrichia
Fusobacteriia
Deltaproteobacteria
Chloroplast
Alphaproteobacteria
Epsilonproteobacteria
Methanobacteria
Flavobacteriia
Sphingobacteriia
Lentisphaeria
Synergistia
Opitutae
Deinococci
Cytophagia
Elusimicrobia
Cyanobacteria
Thermoplasmata
Spirochaetia
Mollicutes
Acidobacteria_Gp4
Acidobacteria_Gp16
Acidobacteria_Gp3
Spartobacteria
Acidobacteria_Gp6
Deferribacteres
Nitrososphaerales
Acidobacteria_Gp10
Halobacteria
Acidobacteria_Gp1
Planctomycetia
Subdivision3
Acidobacteria_Gp7
Thermomicrobia
Gemmatimonadetes
Chlamydiia
Thermoleophilia
Acidobacteria_Gp17
Nitrospira
Armatimonadia

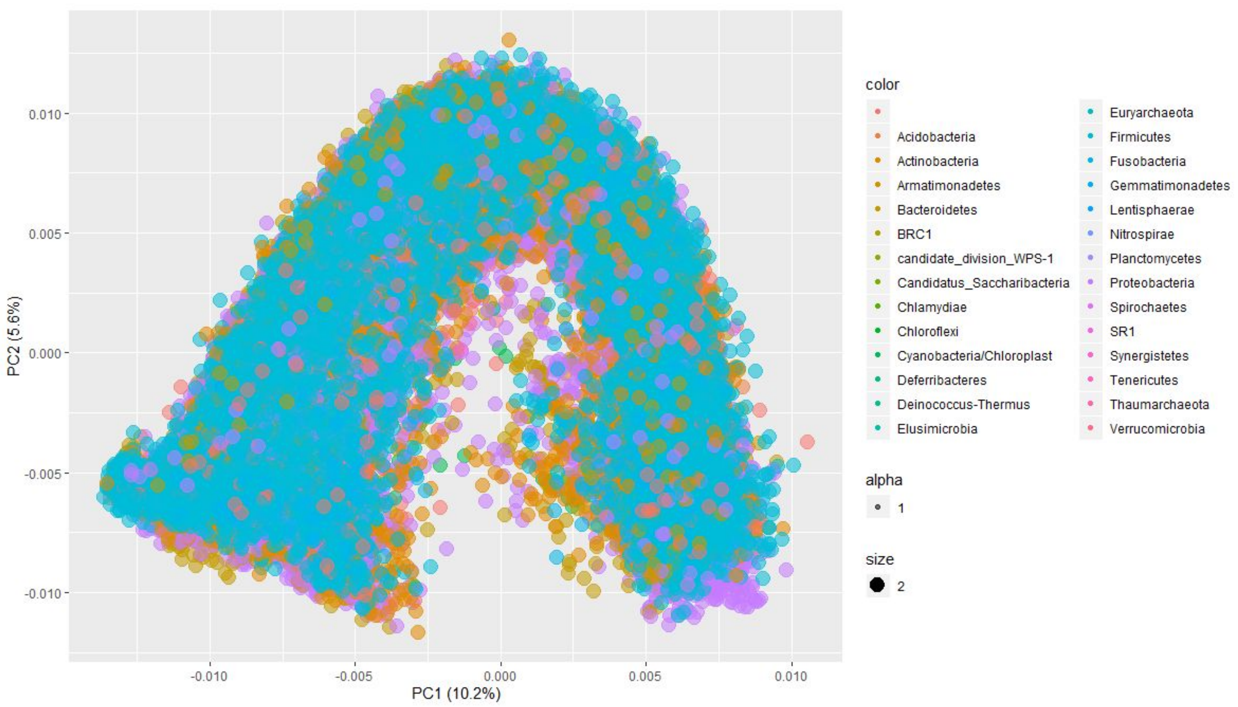

* Section 2.7 is a good way to interpret the properties. Since the authors claims that the properties are actually related to the pathways, more detailed analysis and visualization can help here. Perhaps T-SNE be used to plot/cluster the properties (by using vectors of their correlations to pathways)? Can we see if properties that are similar, have similar metabolic pathways?

Thanks again for this comment. Yes, in fact properties that are similar (based on their taxa vectors) tend to correlate with the same metabolic pathways. To show this, we calculated distances between properties based on both their taxa vectors, and their correlation vectors with pathways. This gave two property x property dissimilarity matrices using the two distance metrics. A mantel test returns a p value of 9.99 * 10^-5 and an r of 0.38. We think this is a reasonably high correlation coefficient, given that this comparison relies on only annotated data, and the fact that expression of genetic capacity varies with environmental context. The results from this test has been added to section 2.7.

In addition, we have included as Supplementary Figure 5 a procrustes analysis that ordinates properties based on their taxa vectors and pathway correlation vectors, and rotates each to minimize the sum of squares between equivalent points in each plot. A permutation test in this context also returns a significant p value of 0.04, and the test is visualized below. Each point is a property position as defined by its pathway correlation vectors under pca. Each arrow points to the equivalent position when a pca is done on properties defined by their taxa vectors. From this, we can see that properties that are similar (in taxa space) tend to correlate similarly to metabolic pathways.

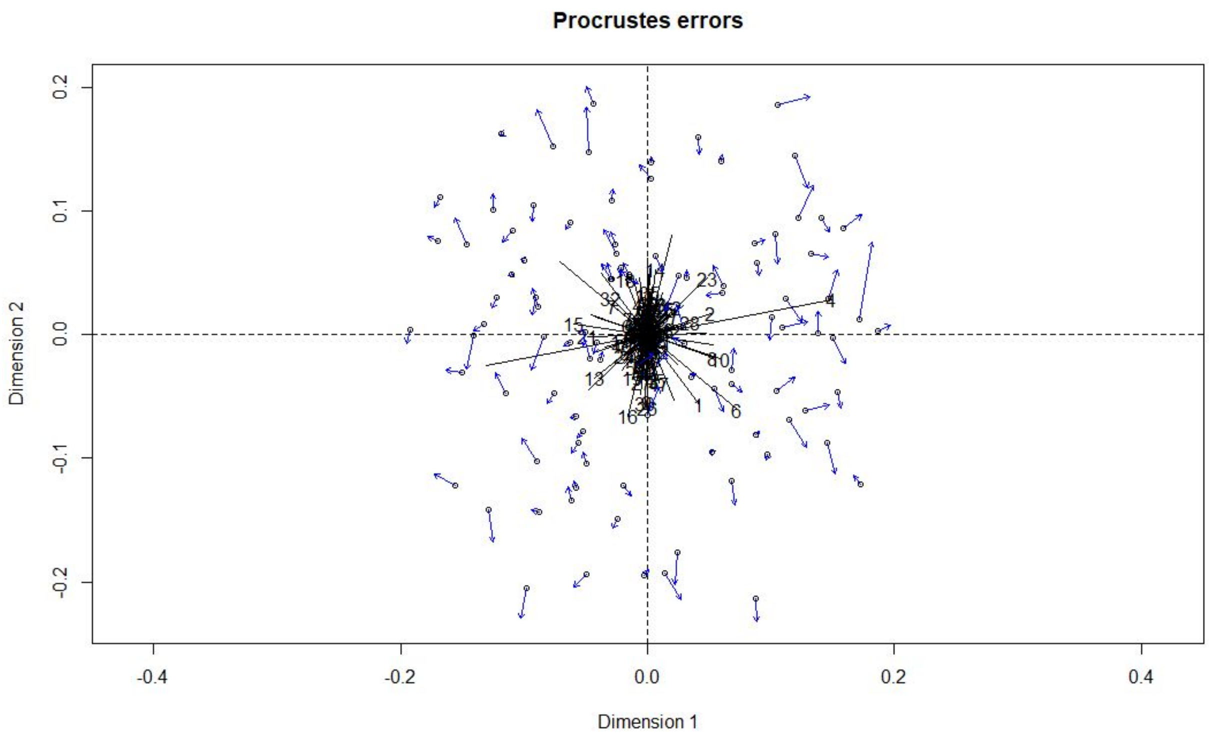

* Can we see if properties that are similar, are more similar taxonomically. The properties might be learning a group of taxa as well.

In order to explore this question in more depth, we performed the hierarchical clustering analysis described above and in figure 6 / section 2.6, figure added below for clarity. Properties are primarily defined by their taxa vectors, and so by construction similar properties are similar taxonomically.

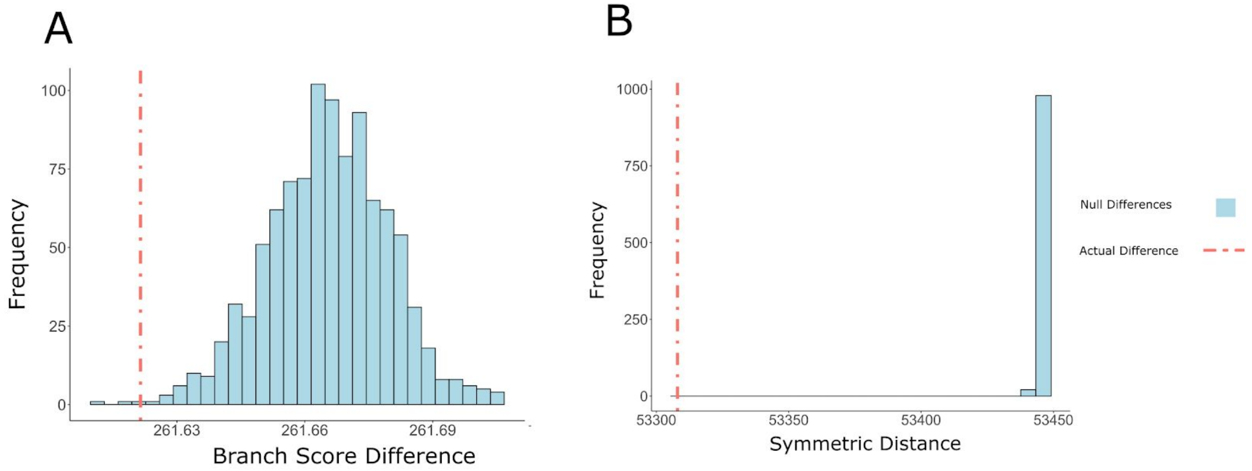

* Figure 1 and section 4.1 is not clear about how the co-occurrence table is made from the ASV table. I think this algorithm should be described in the paper as part of your pipeline. E.g., what

is the dimension of the co-occurrence table? How are the values determined? How is the probability of co-occurrence of taxa i and j with k calculated? Only one sentence (in line 604) talked about the co-occurrence but didn't mention the computation of the probability of co-occurrence.

Thank you for drawing attention to this oversight. These questions are now addressed in sections 4.4.2 and the first paragraph of the Results section. We now explain in 4.2.2 that co-occurrence probabilities between any two taxa i and j is calculated as the sum of all co-occurrences between i and j divided by the number of occurrences of i. A co-occurrence is defined as taxon i and taxon j being observed in the same sample. In section the first paragraph of the results section, we specify that the co-occurrence matrix is 26,726 by 26,726, where 26726 is the number of ASVs present in the American Gut dataset after filtering and quality control.

* Figure 1 and section 4.2 is confusing as there is no 3-dimensional tensor at all in the process. In figure 1, it is correct to have D * C = E. But the intermediate 3D tensor should be removed. The authors can simply describe the averaging idea in 4.2. It looks like D * C becomes a 3-d tensor which is confusing. Also, when applied to a new dataset, what if the dimensions of D and C don't match? Do you drop the columns in C or use the average for those columns?

The 3D tensor has been removed from the figure, and averaging is described in 4.4.2 as suggested.

D is a sample by ASV table, and C is an ASV by property table. In order to take the dot product between D and C, we just need the columns of D to match the rows of C. We've clarified this by updating section 4.4.2, we use BLAST to match the ASV sequences between D and C, and take a 99% similarity cutoff as a hit. This is to account for that fact that most exact ASVs will not be shared between datasets. If an ASV in D does not have a sufficiently close ASV match in C, we drop that ASV. The columns of C are not being manipulated, as these are the embedding dimensions. This explanation has been updated in section 4.4.2 to clarify.

* "The use of ASVs is very confusing. ASV's are meant to improve on OTU thresholding. However, the authors say that they consider any two taxa the same if they have more than 97% identity. This defeats the purpose of ASVs altogether! Since the authors use dada2 pipeline, why not use its results…. Why then use 97% threshold on top of that? The authors need to compare this to using actual ASVs that are not grouped together. Is performance worse when using pure ASVs?"

We heartily agree with the reviewer, which is why we built all the embeddings on ASVs (reproducibility, etc), and apologize for the language being unclear; we never cluster sequences when building the embeddings, we've updated our explanation in section 4.2.2 to clarify this. We do, however, use a threshold when matching sequences across independent datasets. When transforming independent datasets into embedding space, we allowed ASVs to match at 99% (changed from 97) in order to account for some variation across sequencing centers, instruments, and geography. Because the goal of this study was to leverage public datasets to inform studies from different sequencing centers, we've decided to make it as translational as

possible by allowing the inexact matching. Supplementary Figure 1 now shows the analysis from Figure 4 using a 97% and 100% similarity threshold instead of 99%, for the sake of comparison. Supplementary Figure 2 equivalently shows the analysis from figure 5 using 97% and 100% similarity thresholds for two independent datasets.

* Can you sweep the dimensions and find a similar plot to Fig. 2 when using actual ASVs and not those thresholded to 97%

Yes, figure 2 is made using embedded ASVs only (no clustering), as it was made with the AGP dataset. We apologize for the confusion, and edited the text as indicated in comment 8 above.

* The author mentioned that the counts were normalized by an inverse hyperbolic sin function. Is this for raw count input data only (since this sentence is right after line 523 where the authors listed all three possible input formats to the RF model)? If so, why not relative abundance or maybe centered log ratio transform?

The inverse hyperbolic sin function, $sin^{-1}(x) = log(x + (x^2 + 1)^{1/2})$, was selected because it mimics the function $log(2x)$ function almost exactly, except for behavior near 0 (59). Below 1, log will return a negative value, and at 0, log is undefined. In contrast, inverse hyperbolic sin does not fall below 0 when the argument is low, and is defined as 0 at 0 (1).

This normalization was used on raw count input data. Normalized count data was then used as was, transformed using PCA, and embedded. So all transformations were made after raw count normalization. Section 4.4.1 has been modified to clarify.

* In Figure 3B2 and Figure3B3, the authors have GloVe embedding data and PCA data in 113 dimensions (which after reading the methods is the 100 properties plus 13 sample-associated metadata features). This is consistent with the text. However, for Non-reduced data, in Fig 3B1, it says that 26726 features are used but in the text, it says that 26739 features are used. What is true here? Were 26726 or 26739 features used for the non-reduced RF model for training?

Thank you for pointing out this discrepancy. 26739 features were used, and this has been modified in figure 3.

* The transition between first several sections in results section (2.1, 2.2, 2.3) seems not smoothing. Maybe in the Result section, first give an overview of the data and target (problem setup), then get to the specific results.

Thank you for this suggestion. The following paragraph has been added to the beginning of the Results section as an overview: “By applying the GloVe algorithm to 18,750 gut microbiome samples from the American Gut Project, we construct a 26726 ASV by 100 property embedding transformation matrix, which can be used to project any sample by ASV table into embedding space. We then test the performance of models trained on taxonomic counts, embedded data, and pca transformed data across combinations of training/testing datasets: 1. Models trained and tested on the same dataset used to construct embeddings (AGP) 2. Models trained and tested on an independent IBD dataset (Halfvarson) and 3. Models trained on one dataset (AGP) and tested on an independent set (Halfvarson). We find that models trained on

embedded data perform as well or better than models trained on ASV count data despite using 250 fold fewer variables, and far outperformed models trained on PCA reduced data. We find that phylogeny and metabolic functional capacity both exhibit significant correlations with property structure."

* Figure 7 is upside down.
Thank you for pointing out the oversight, it is fixed.

* In line 497, the link to the github repo is 404 NOT FOUND. I only found https://github.com/MaudeDavidLab/embeddings_final (please update the link in your manuscript). The README could provide explicit examples instead of vague statements. Instead of "Run Piphillan", can you give the exact command that was run? Can you give a toy example of a dataset that someone may want to run and then step-by-step exact commands that show how to run the whole pipeline?

The link is updated, thanks for pointing this out. We have indicated more details on how to run this pipeline with name the specific commands to run, and more detailed instructions on what task each script does, and what files they output.

* Organization of the paper can be improved. Ideas are provided in different sections in a fragmented way: First 2 paragraphs of section 3.1 are the explanation of the employed method. So, they can be moved to the Materials and Methods section. Third and fourth paragraphs of section 1.3 are also explaining the idea of the connection between words and how properties capture these connections. These paragraphs can be moved to the method section.

Thank you for pointing out this inconsistency. The two paragraphs mentioned from section 3.1 have been moved to the methods section as suggested, and the third and fourth paragraphs of section 1.3 have been moved to methods and removed, respectively.

* Page 19, Section 4.1 line 502 and 503: "In this algorithm, the metric to be preserved is a function of P_ik / P_jk, the probabilities of co-occurrence of taxa i and j with k." is ambiguous. The notation implies that P_ik is divided by P_jk.

The notation has been clarified in 4.3. It now reads $\frac{P_{ik}}{P_{jk}}$

* Figure 7: The Caption of Figure 7 says "Each column in each heat map represents a metabolic pathway from KEGG e.g.321 ko00983). Each row is a dimension in either GloVe or PCA embedding space". This means that in the graph each point represents a correlation coefficient. However, in the figure, both labels are provided for the y-axis (on either side). Based on the caption, "metabolic pathway" is x-axis, not y-axis. Also, a legend that explains the numerical values of color codes can improve the figure.

Thank you for pointing out the missing legend, one has been added. Metabolic pathway is labeled on the x axis, to show that each column (x coordinate) is a metabolic pathway. Dimension is labeled on the y axis, to show that each row (y coordinate) is a dimension. We believe this labeling is consistent with the text.

* Figure 6: Caption of figure 6 says “lighter color signifies a higher density of taxa pairs” But the unit of density is not mentioned in the legend. Mentioning the unit will make the figure more specific and informative.

Thank you for your comment. The text should read “higher count of taxa pairs”, and has been changed in the figure legend.

* Figure 4 B1, B2: Markers should be added to the plots

We have updated Figure 4 following your recommendation.

* In line 305, Piphillan is misspelled as Piphillian

Thank letting us know, it has been fixed

* First of all, the authors made lots of hand-waving claims of superiority of this invention and how it will reframe "the microbiome analysis mindset" (line 49) or "shift the analysis paradigm" (line 173). I am highly skeptical about these statements. I suggest that the authors focus on science not marketing.

Thank you for this feedback. The specific statements in question have been removed, and the tone of the manuscript as a whole as been shifted towards the benefits of utilizing public dataset information.

*Second, the abstract and introduction are unnecessarily lengthy. This reduced my joy in reading this manuscript. Some paragraphs are just explanations of terms. For example, the whole section 2.1 is introducing AUROC and AUPR. I doubt if readers of PLOS Computational Biology would need this basic information.

Thanks again for this feedback. The abstract and introduction have been made more concise. The explanations of AUROC and AUPR have been removed.

*Line 125: "several studies have attempted to integrate phylogenetic..." This sounds like few people are using phylogeny. But in contrast, technologies like UniFrac and Faith's PD have been massively used for more than a decade.

We agree that the wording of this phrase does not give credit to the ubiquity of phylogenetic metrics, and apologize for the lack of clarity with this statement, which was meant to provide a transition into the concept of using phylogenetic information not as a metric like UniFrac or Faith’s PD, but rather as a feature transformation, as was done by Wolosynec et. al. The sentence in question has been changed to: “Patterns in 16S sequence relatedness have been used for feature transformation as well.”

*Line 89: "More specifically, predictive models that differentiate between disease and healthy guts based on microbiome composition in one dataset can rarely be successfully applied to samples from the same patient population collected independently." I doubt it.

Thanks for your feedback, we have removed the claim.

*The author's understanding of the "current" trend of differential abundance testing is outdated (e.g., citations 32 and 33). The authors repeatedly indicated that current researchers rely on the observation of differential abundance of individual taxa, rather than caring the overall image of the community. This is very exaggerated.

We agree that current research does care greatly about the overall image of a community, and so have removed the language indicating that commonly employed techniques assume independence of bacterial species, including the indicated citations.

*The authors indicated that abundance-based taxon filtering, taxon categorization and binning are forms of "dimensionality reduction" (line 106). I think most statisticians will not agree. Instead, what microbiologists usually refer to as "dimensionality reduction" include beta diversity distance matrices and multi-dimensional scaling techs based on them, such as PCoA and NMDS. I am surprised that these two terms were not mentioned at all in this manuscript, but instead the authors extensively discussed PCA. It was known that PCA on taxon counts does not yield as informative signals than PCoA on more deliberate beta diversity distance matrices.

Thank you for the helpful comments. It’s true that we mention only PCA and MDS as examples of ordination. PCoA and NMDS are now specifically mentioned in section 1.2

It is true that most microbiologists normally only think of ordination techniques like PCoA and NMDS as dimensionality techniques. To my understanding, however, dimensionality reduction techniques can take on two forms: feature extraction/transformation and feature selection(2). The ordination techniques mentioned in the comment (PCoA, NMDS) and agglomeration by taxonomic relationship, fall under the category of feature transformation, while abundance-based taxon filtering is considered feature selection. Both feature transformation and feature selection are considered dimensionality reduction techniques (3). We have edited section 1.2 to clarify.

*Also for the author's information: Recent years have seen a new trend of differential abundance testing that is related to the authors' idea of using co-occurrence patterns. That is, to rely on the log-ratios among taxa instead of the relative abundance of individual taxa. I hope the authors can make some comments on those methods.

Thank you for your comment and pointing out log-ratios based analysis. Our paper is definitively not the first to consider using co-occurrence patterns in the analysis of microbiome data, as co-occurrence networks and related technologies have long been a staple of the field.

We have added in section 1.1 an indication that the literature has been utilizing log-ratios for the analysis of microbiome dataWe mention the technique of comparing the log-ratios of all pairs of taxa (4), which identifies differences in the relationship of taxa pairs between groups:

“The address [the problem of related taxa pairs], it is often appropriate to base analysis on the log-ratios of pairs of taxa, which allows identification of differences in the relationship of taxa pairs between groups.”

*The test on Halfvarson data is great. But one single case study does not make a strong argument. Can the authors test some other datasets? Just a suggestion: Along with AGP, the Qiita platform hosts a large number of public 16S V4 datasets, and many of them were

generated using a uniform protocol (the "EMP protocol"). This ensures low bias in cross comparison / meta analysis.

We repeated the analysis involving training a model on AGP data and testing on an independent set with a second independent dataset created by Schirmer et. al, and have included these results in Figure 5. We optimize model hyperparameters for maximum AUC under the precision-recall curve using cross-validation of the AGP data. We find that the ASV based model performs very poorly on the second independent dataset, while the embedding-based model performs well.

*What is "normalized taxonomic count data"? The authors stated somewhere (line 526) that "Counts were normalized by applying an inverse hyperbolic sin function". This is not how microbiologists normalize their taxon tables, to my knowledge. Can the authors explain the rationale?

Thank you for the suggestion to include more justification for normalization choice. We have added this justification in section 4.4.1, and copy it here: “The inverse hyperbolic sin function, $sin^{-1}(x) = log(x + (x^2 + 1)^{1/2})$, was selected because it mimics the function $log(2x)$ function almost exactly, except for behavior near 0 (59). Below 1, log will return a negative value, and at 0, log is undefined. In contrast, inverse hyperbolic sin does not fall below 0 when the argument is low, and is defined as 0 at 0 (1).”

19. Mantel r = 0.12 is quite low. The significant p-value is likely due to the large sample size (18,000). This section (2.6) overall does not sound like very strong evidence.

We agree with the reviewer that the correlation reported here is quite low, and diffuses the message of the paper. This test was intended to test whether the phylogenetic distance metric and distance in embedding space were in any way correlated, we have changed the text to better explain this in section 2.6 and Supplementary Figure 3 legend. Because this is not the main purpose of our paper, and phylogeny seems to be a weak but not major driver of taxa co-occurrence patterns, we have decided to move this analysis to be supplementary figure 3, and have replaced figure 6 and section 2.6 with a different analysis that shows the relationship between properties and phylogeny (see below) which was suggested by the other reviewer. We compare the tree structures between a phylogenetic tree and a tree built using hierarchical clustering of taxa based on their properties. We then perform a permutation test with 1000 permutations to demonstrate that the trees are significantly similar to each other in topology and branch length distances.

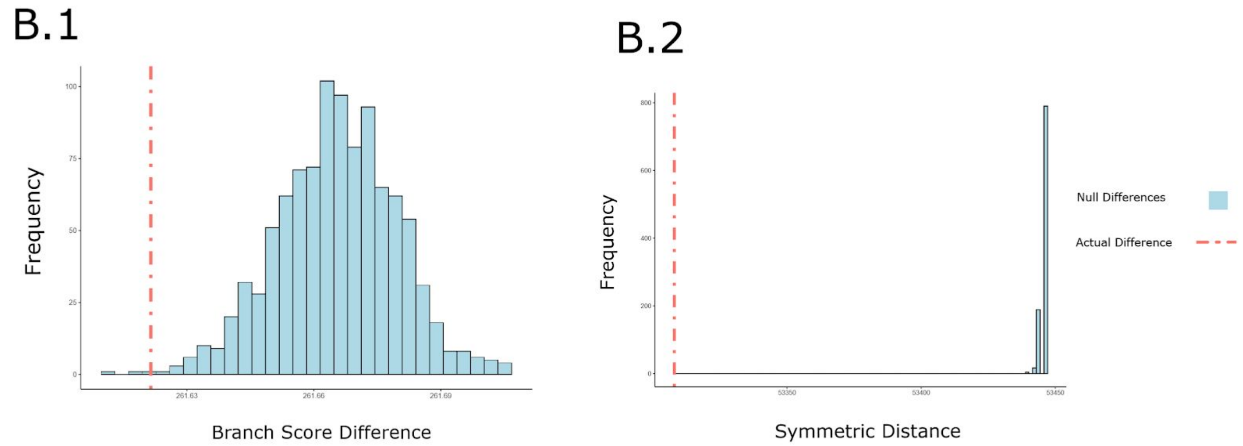

Finally, one trivial thing also affected my reading experience: The singular form of "taxa" is "TAXON"! Please correct it.

Thanks for pointing this out, it is fixed. In most places, taxa was actually replaced with ASV for clarity.

*Final of finally, the source codes are available at GitHub, but they do not look like in the form that users can easily take advantage of. It seems to me that the authors simply dumped codes there to meet the journal's requirement. If the authors really plan to call for a paradigm shift in the field, they should spend some time on software engineering so that more people will come and experience this new method.

Thank you so much for your suggestion. We have greatly improved the available code and pipeline, and updated the workflow. Please see the updated workflow.md in the github that provides reproducible steps in an easier manner.

Additional comments: We performed additional benchmarking experiments as requested by the reviewer. We have modified figures 4 and 5 to provide more information, and changed the recommended similarity threshold for matching ASVs across datasets to 99%. In figure 4, results are now averaged over 50 random seeds which represents a more robust analysis. In figure 5, we report performance over 2 datasets, optimize hyperparameters for AUC under the precision recall curve, and include the same analysis at 2 other similarity thresholds (97% and 100%) in supplementary figures 1 and 2.

REFERENCES:

1. Burbidge JB, Magee L, Robb AL. Alternative Transformations to Handle Extreme Values of the Dependent Variable. J Am Stat Assoc. 1988;83(401):123–7.
2. Tang B, Shepherd M, Heywood MI, Luo X. Comparing Dimension Reduction Techniques for Document Clustering. In: Kégl B, Lapalme G, editors. Advances in Artificial Intelligence. Berlin, Heidelberg: Springer; 2005. p. 292–6. (Lecture Notes in Computer Science).
3. Wei H, Billings SA. Feature Subset Selection and Ranking for Data Dimensionality

Reduction. IEEE Trans Pattern Anal Mach Intell. 2007 Jan;29(1):162–6.
4. Calle ML. Statistical Analysis of Metagenomics Data. Genomics Inform [Internet]. 2019 Mar 31 [cited 2019 Dec 20];17(1). Available from: http://genominfo.org/journal/view.php?number=549
5. Mandal S, Van Treuren W, White RA, Eggesbø M, Knight R, Peddada SD. Analysis of composition of microbiomes: a novel method for studying microbial composition. Microb Ecol Health Dis. 2015;26:27663.
