# Supplementary material for: Decoding the language of microbiomes using word-embedding techniques, and applications in inflammatory bowel disease
Source: PLoS Comput Biol. 2020 May 4;16(5):e1007859. doi: 10.1371/journal.pcbi.1007859 (PMC7244183; doi:10.1371/journal.pcbi.1007859)
Supplement: Attachment — Submitted filename: reviewer_comments_round_2.docx [file pcbi.1007859.s012.docx]

1. I think the authors didn’t address our comments on finding species that are co-occurring. The authors complained that they can’t extract new information from their taxa-taxa co-occurrence network made by embedding vectors. But in fact, the authors can just do case studies in a few interesting species instead of plotting everything all at once in phylum level (which is actually very high level). For example, the authors can simple go to the OTU table and extract species that have high co-occurrence score (both have high abundance for different samples). Then just look at them. See you find species A and B are co-occurring and species C and D are co-occurring and species E and F are probably competing (one exist then the other doesn’t) Then in embedding space, you can plot the embedding of those species and see if you can discover the relationship in the embedding space. You might even be able to interpret some of the properties (dimensions in the embedded vector).

Thank you for your suggestion, the following information has been added to section 2.6. We’ve followed this recommendation by adding the plot below as Fig.6B (complete figure pasted below), a 2D PCoA ordination using cosine distance of 1500 randomly selected taxa in embedding space. 10 pairs of ASVs were selected based on their high spearman correlation in the American Gut ASV table across all samples(r > 0.7). Each pair is denoted by a different shape. Color shows the family classification of the selected pairs, and all other ASVs are shown in grey. It should be noted that 5 out of the 10 pairs share a family while 5 do not. Using this plot, we first validate that the method is performing as we expect - pairs of taxa that directly co-occur are also close in embedding space. The plot also visualizes pairs of ASVs that directly co-occur, implying that there may be some synergy between the pairs.

We also find that ASV pairs with the highest spearman correlations of counts across samples are generally close in embedding space, as expected. Of the 10 annotated ASV pairs with highest spearman correlation, we find 5 share a family while 5 do not (Fig 6. B). To further this analysis, we also identified 3 pairs of ASVs that were close together in embedding space, but *did not strongly* directly correlate in raw counts. These ASVs are more likely to share a similar biological function and potentially even compete. The pairs include *Bacteroides vulgatus* and *Escherichia coli, Bacteroides uniformis* and *Faecalibacterium prausnitzii,* and *Faecalibacterium prausnitzii* and *Eubacterium rectale*.

We have also added the following section 3.4 to the Discussion to further explore those results:

Results from section 2.6 suggest that phylogeny does drive property structure to some extent, as might be expected. Using embeddings, we identified 3 genera, *Streptococcus*, *Staphylococcus*, and *Neisseria*, that may interact synergistically as their members exhibit similar patterns to each other in embedding space and also frequently co-occur directly. Literature does not suggest that the most heavily studied members of these genera are the pathogens *S. aureus, S. pneumoniae, and N. meningitidis* directly interact, but little is known about the other members of these genera. We also identified 3 pairs of ASVs that are close in embedding space but *do not* co-occur directly; we hypothesize these microbes may therefore share similar ecological functions. The first pair is annotated as *Bacteroides vulgatus* and *Escherichia coli*. Both these species are known to cause gastrointestinal inflammation and colitis, and have not been reported in the literature to depend on one another, suggesting these microbes may fill the same niche in the human gut [(44)](https://www.zotero.org/google-docs/?F2F56h). The second pair is *Bacteroides uniformis* and *Faecalibacterium prausnitzii*. These species are on the short list of ‘next generation’ probiotics that yielded positive outcomes for inflammatory and metabolic disorders, potentially implying their similar functions [(45,46)](https://www.zotero.org/google-docs/?8JHiVm). The third pair is *Faecalibacterium prausnitzii* and *Eubacterium rectale*. These species are heavy butyrate producers, making it possible that they perform similar ecological functions in the human gut [(47)](https://www.zotero.org/google-docs/?IQMQhz).

2. As for t-SNE and PCA plots. Yes, if you are dealing a lot of taxa, it is very hard to visualize. But what if you just do a subset? For example, plot all the genera in a few families? Then we should expect that similar genera or families cluster together and distance from other families/genera?

To answer the reviewer’s comment, we have included Fig.6C. The plot is a 2D t-SNE ordination of embedding space with ASVs colored by genus. 5000 ASVs were randomly selected to represent the space, and 13 genera were selected to be colored. In particular, the three genera Staphylococcus (green), Streptococcus (pink), and Neisseria (yellow) appear close together with similar trajectories in this space and do not appear elsewhere, implying that these genera may either interact directly or share some similarities.

A PCoA ordination (Supplementary Figure 3) using correlation of normalized counts (not embedding space) shows that these 3 genera do often occur in direct correlation with one another. This potentially implies that these microbes from these genera may interact synergistically.

Supplementary Figure 3:

Figure 6

3. Section 2.7 has been improved a lot. However, the procrustes analysis is not clearly explained in the method section in the paper. Like I am not certain what “ordinates properties based on their taxa vectors and pathway correlation vectors" -- what are the exact vectors being input into the Procrustes analysis? What do the long vectors out of the origin mean.. and why are they given numbers and not the shorter arrows outside? In the end, I’m not sure what this figure is showing.

The procrustes analysis was meant as an additional way to visualize the Mantel test reported in section 2.7. Given that this analysis does not bring additional information, and given your feedback, we propose to remove Suppl Figure 5 completely.

4. * The authors use “et. al” and not “et al.”

Thank you for pointing this out, we have fixed it.

5. I would say that Supplementary Fig. 3 is still confusing and should be removed.

Thank you for the feedback, it has been removed.
